# Overexpression of S100A9 in tumor stroma contribute to immune evasion of NK/T cell lymphoma and predict poor response rate

**DOI:** 10.1038/s41598-021-90794-3

**Published:** 2021-05-27

**Authors:** Zhiyuan Zhou, Xinfeng Chen, Zhaoming Li, Xinhua Wang, Mingzhi Zhang

**Affiliations:** 1 Department of Oncology, The First Affiliated Hospital of Zhengzhou University, No. 1 Jianshe East Road, Zhengzhou, 450000 China; 2 Institute of Clinical Medicine, The First Affiliated Hospital of Zhengzhou University, No. 1 Jianshe East Road, Zhengzhou, 450000 China; 3 Biotherapy Center, The First Affiliated Hospital of Zhengzhou University, No. 1 Jianshe East Road, Zhengzhou, 450000 China

**Keywords:** Cancer, Oncology

## Abstract

NK/T cell lymphoma (NKTCL) represents an aggressive lymphoid malignancy characterized by dismal prognosis. Immune-checkpoint blockade has shown promising efficacy in NKTCL. However, the molecular mechanisms underlying immune evasion in NKTCL have never been explored. Here, proteomic analysis was used to identify the differentially expressed proteins between NKTCL patients and healthy individuals. We found that S100A9, an immunosuppressive molecule, was much higher in NKTCL patients both in serum and tumor stroma. Elevated level of S100A9 was associated with advanced stage, poor overall response and early recurrence. Moreover, percentage of myeloid-derived suppressor cells (MDSCs) in peripheral blood was positively correlated with levels of S100A9. Low concentration of S100A9 promoted proliferation of NKTCL cells, while did not affect cell apoptosis and cell cycles. Furthermore, programmed death ligand 1 (PD-L1) expression on NKTCL cells was up-regulated by S100A9 through activation of ERK1/2 signaling. Inhibition of ERK1/2 signaling significantly decreased tumor growth and PD-L1 expression induced by S100A9. In conclusion, our research firstly identified S100A9 as an immune suppressor in the tumorigenesis of NKTCL via accumulation of MDSCs and upregulation of PD-L1 expression. S100A9 may serve as a potential target to increase the efficacy of immunotherapy in NKTCL.

## Introduction

NK/T cell lymphoma (NKTCL) is a distinct subtype of non-Hodgkin lymphoma characterized by aggressive course and dismal survival. Populations from China, Japan and South America are more frequently affected, while the incidence rate is low in western countries^1^. Most patients present primary lesions in the nasal cavity, and occasionally in lung, skin and intestinal tract^2^. Recent researches indicated that activating mutations of DDX3X, JAK3 contribute to the pathogenesis of NKTCL^3,4^. EBV infection and inflammation are also deeply implicated in the development of NKTCL. EBV-encoded oncoprotein latent membrane protein 1 (LMP-1) and miRNA-BARTs lead to proliferation of NKTCL tumor cells^5,6^. Moreover, most NKTCL patients have long history of chronic rhinitis. Tumor tissues are usually infiltrated with abundant immune and inflammatory cells, which may secrete cytokines into tumor microenvironment and peripheral blood to accelerate tumor progression and immune escape. However, currently the key factors and molecular mechanism underlying have not been well elucidated in NKTCL.

For localized NKTCL, most patients could benefit from L-asparaginase-based chemotherapy in combination with radiotherapy^7^. High-dose chemotherapy such as SMILE regimen have showed promising response rates in advanced NKTCL patients^8^. We also reported the remarkable efficacy of DDGP regimen (gemcitabine, pegaspargase, cisplatin and dexamethasone) in the treatment of advanced NKTCL patients^9^. Unfortunately, some patients still experience therapeutic failure due to primary or acquired resistance. Immune checkpoint inhibitors targeting PD-1/PD-L1 axis have been demonstrated effective in certain cancers such as melanoma and lung cancer^10,11^. Overall response rate (ORR) of 100% was obtained in relapsed/refractory NKTCL patients treated with pembrolizumab^12^. Uncovering the upregulation mechanisms of PD-L1 in NKTCL would improve efficacy of immunotherapy. EBV-encode LMP-1 was reported to be able to activate NF-KB signaling and increased PD-L1 expression in NKTCL cells^13^. Increasing evidences suggested the role of inflammation in regulating PD-L1 expression. Macrophage secreted TNF-α enhanced PD-L1 expression through increasing COP9 signalosome 5 expression in breast cancer^14^. However, other molecules regulating PD-L1 expression in NKTCL need to be explored.

In the present study, we conducted proteomic analysis to identify the differentially expressed proteins in serum from NKTCL patients and healthy individuals. Among the dysregulated proteins, S100A9 was confirmed to be elevated in both serums and tumor stroma from NKTCL patients. Moreover, clinical analysis implied that high expression of S100A9 in tumor stroma predicted advanced stage, poor response rate and early recurrence. Furthermore, S100A9 protein promoted proliferation and increased PD-L1 expression in NKTCL cells via activation of ERK1/2 pathway. S100A9 may mediate inflammation-associated NKTCL and function as a novel target to improve the management of NKTCL patients.

## Results

### NKTCL patients displayed higher levels of S100A9 in serum and tumor stroma than healthy individuals

**Figure 1:**
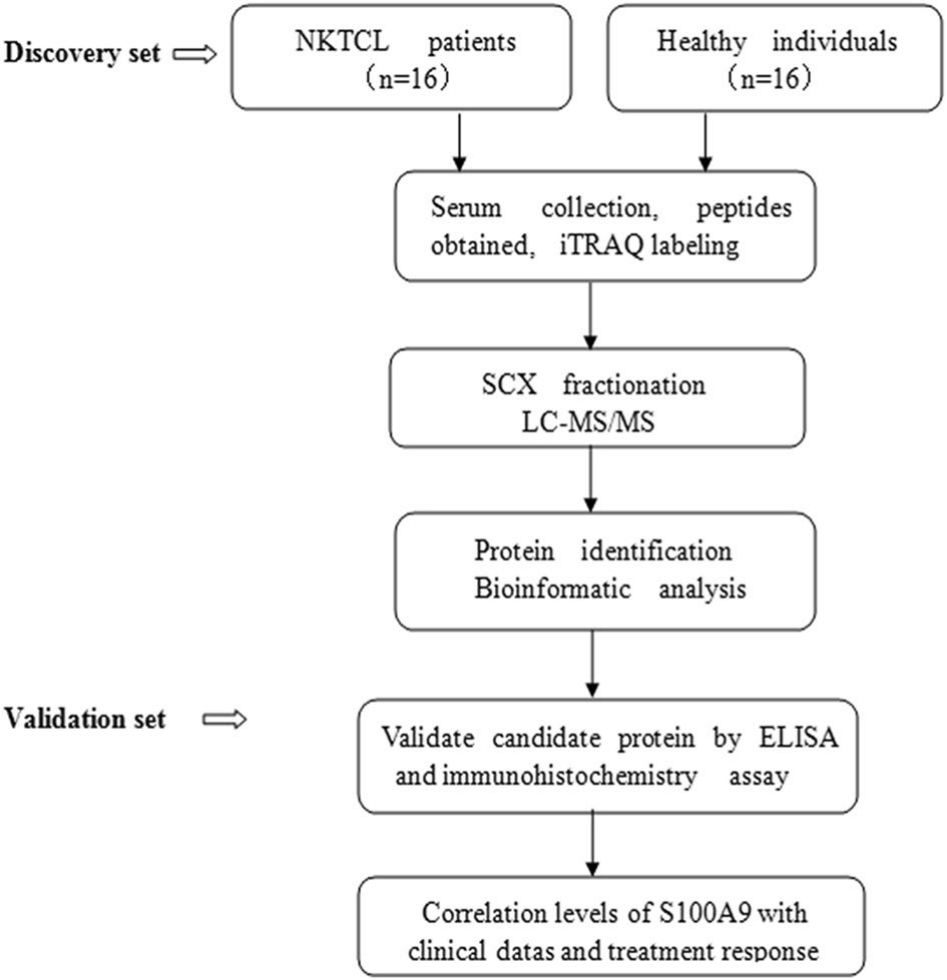
Work procedure for proteomic analysis to identify the differentially expressed proteins in serum between NKTCL patients and healthy individuals.

**Figure 2:**
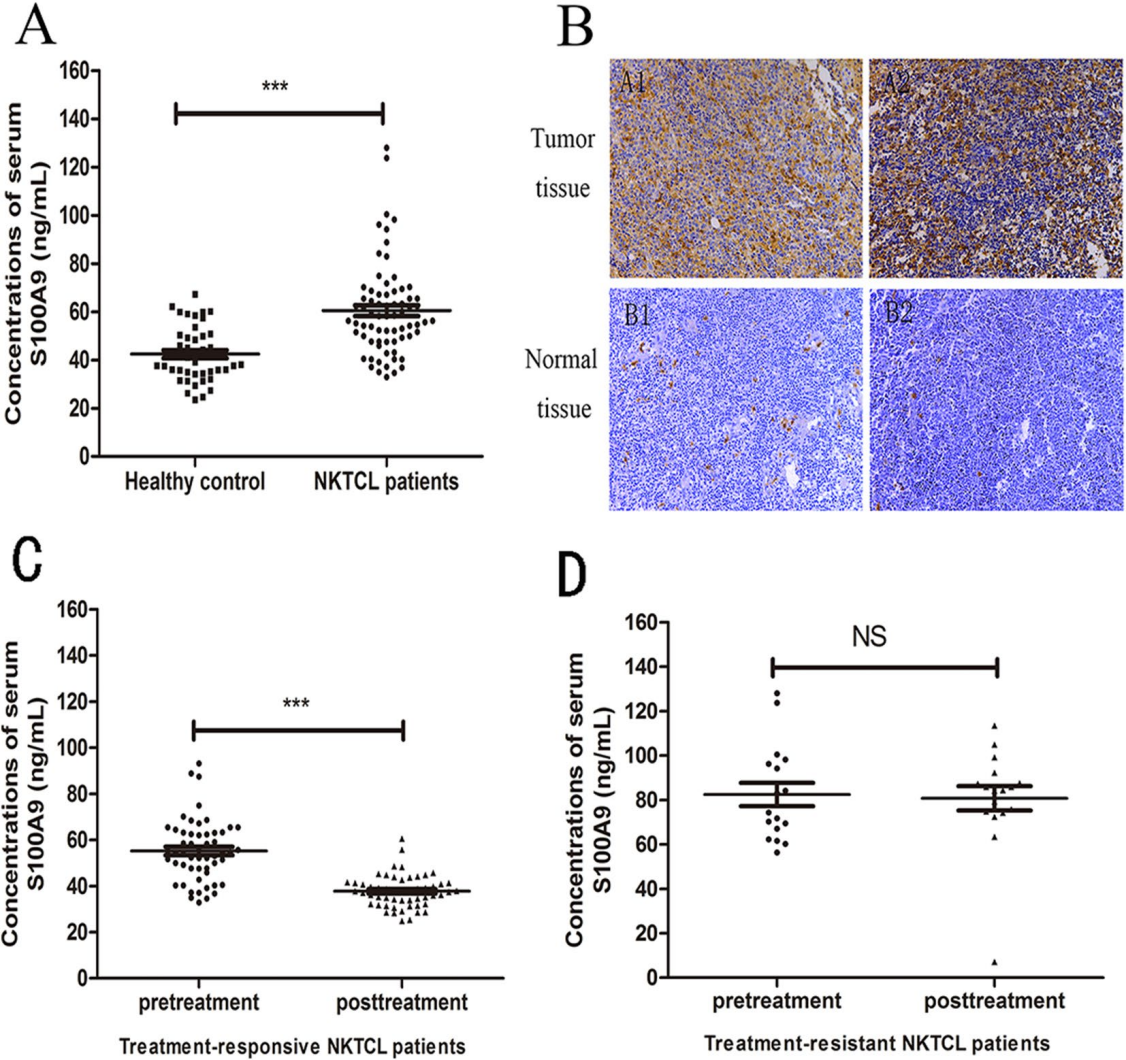
Validation of candidate protein S100A9 in NKTCL patients and healthy individuals. (**A**) NKTCL patients displayed higher serum levels of S100A9 than healthy individuals (*p* < 0.001). (**B**) Normal nasal tissues did not express S100A9 protein, while S100A9 was highly expressed by tumor stromal cells in NKTCL tumor tissues. (**C**) Posttreatment levels of S100A9 declined dramatically compared with pretreatment levels in chemo-sensitive patients (*p* < 0.001). (**D**) No significant change was observed between pretreatment and posttreatment levels of S100A9 (*p* > 0.05). ***p < 0.001, **p < 0.01, NS *p* > 0.05.

We conducted comparative proteomic analysis to identify the differentially expressed proteins in serum between NKTCL patients and healthy individuals (work procedure shown in Fig. 1). Compared with healthy individuals, a total of 109 upregulated and 82 downregulated proteins were identified in NKTCL patients (Supplemental Table 1). Among the upregulated proteins, S100A9, an immune-related molecule, has been reported to play a key role in tumor progression and immune escape. Therefore, we selected S100A9 as a candidate protein and validated its expression in NKTCL patients and healthy individuals, respectively. ELISA assay further confirmed that NKTCL patients presented higher levels of serum S100A9 than healthy individuals (*p* < 0.001) (Fig. 2A). In addition, we examined S100A9 expression in NKTCL tumor tissues. Immunohistochemical staining showed that normal nasal mucosa tissues did not express S100A9, while S100A9 was highly expressed by tumor stromal cells in NKTCL (Fig. 2B). Moreover, we found that in chemo-sensitive patients, posttreatment serum levels of S100A9 dramatically declined (*p* < 0.001) (Fig. 2C), while for chemo-resistance patients levels of S100A9 did not altered (*p* > 0.05) (Fig. 2D).

### Elevated levels of S100A9 was associated with advanced stage, poor response rate and early recurrence in NKTCL patients

To investigate the clinical relevance of S100A9, NKTCL patients were divided into high-S100A9 group and low-S100A9 group according to serum levels of S100A9. Receiver-operating-characteristics (ROC) analysis was employed to determine the S100A9 cutoff for high-S100A9 and low-S100A9. Serum levels of S100A9 at the concentration of 62.0 ng/ml possessed a better prognostic value and discriminatory threshold value. Serum levels of S100A9 were further correlated with clinical characteristics and treatment outcome. There was no significant difference between two groups in regard of age and sex. We found that there was no significant differences between high-S100A9 group patients and low-S100A9 group patients in regard of EBV-DNA copies, PINK-E score and treatment strategies. Higher S100A9 expression was associated with advanced disease stage (*p* < 0.05) (Table 1). Moreover, patients with high levels of S100A9 predicted inferior overall response rate (ORR) than low-S100A9 patients (30.4% vs 69.6%, *p* = 0.021). Patients with higher expression of S100A9 also predicted early recurrence rate (61.1% vs 38.9%, *p* = 0.015) (Table 1).

**Table 1:** Clinical relevance of serum S100A9 with pathological characteristics, response rate and early recurrence in NKTCL patients.

Variables	Total (N)	High-S100A9 n = 22	Low-S100A9 n = 36	*P* value
Age				0.757
≤ 60	49	19 (38.8%)	30 (61.2%)	
> 60	9	3 (33.3%)	6 (66.7%)	
Sex				0.739
Female	20	7 (35%)	13 (65%)	
Male	38	15 (39.5%)	23 (60.5%)	
B symptom				0.639
Negative	32	13 (40.6%)	19 (59.4%)	
Positive	26	9 (34.6%)	17 (65.4%)	
IPI score				0.356
≤ 2	41	14 (34.1%)	27 (65.9%)	
> 2	17	8 (47.1%)	9 (52.9%)	
Stage				0.023
localized	37	10 (27.0%)	27 (73.0%)	
Advanced	21	12 (57.1%)	9 (42.9%)	
Treatment response				0.021
ORR (CR + PR)	46	14 (30.4%)	32 (69.6%)	
SD + PD	12	8 (66.7%)	4 (33.3%)	
EBV-DNA copies				0.517
< 5000 copies/ml	42	17 (40.5%)	25 (59.5%)	
≥ 5000 copies/ml	16	5 (31.2%)	11 (68.8%)	
PINK-E				0.587
≤ 2	37	15 (40.5%)	22 (59.5%)	
> 2	21	7 (33.3%)	14 (66.7%)	
Treatment strategy				0.622
Chemotherapy	24	10 (41.7%)	14 (58.3%)	
Chemotherapy with radiotherapy	34	12 (35.3%)	22 (64.7%)	
Early recurrence				0.015
No	40	11 (27.5%)	29 (72.5%)	
Yes	18	11 (61.1%)	7 (38.9%)	

*IPI* international prognostic index, *ORR* overall response rate, *CR* complete response, *PR* partial response, *SD* stable disease, *PD* progression disease, *EBV* Epstein-barr virus, *PINK-E* prognostic index of natural killer lymphoma-Epstein-barr virus.

### Percentage of myeloid-derived suppressor cells (MDSCs) in peripheral blood was positively correlated with levels of S100A9

**Figure 3:**
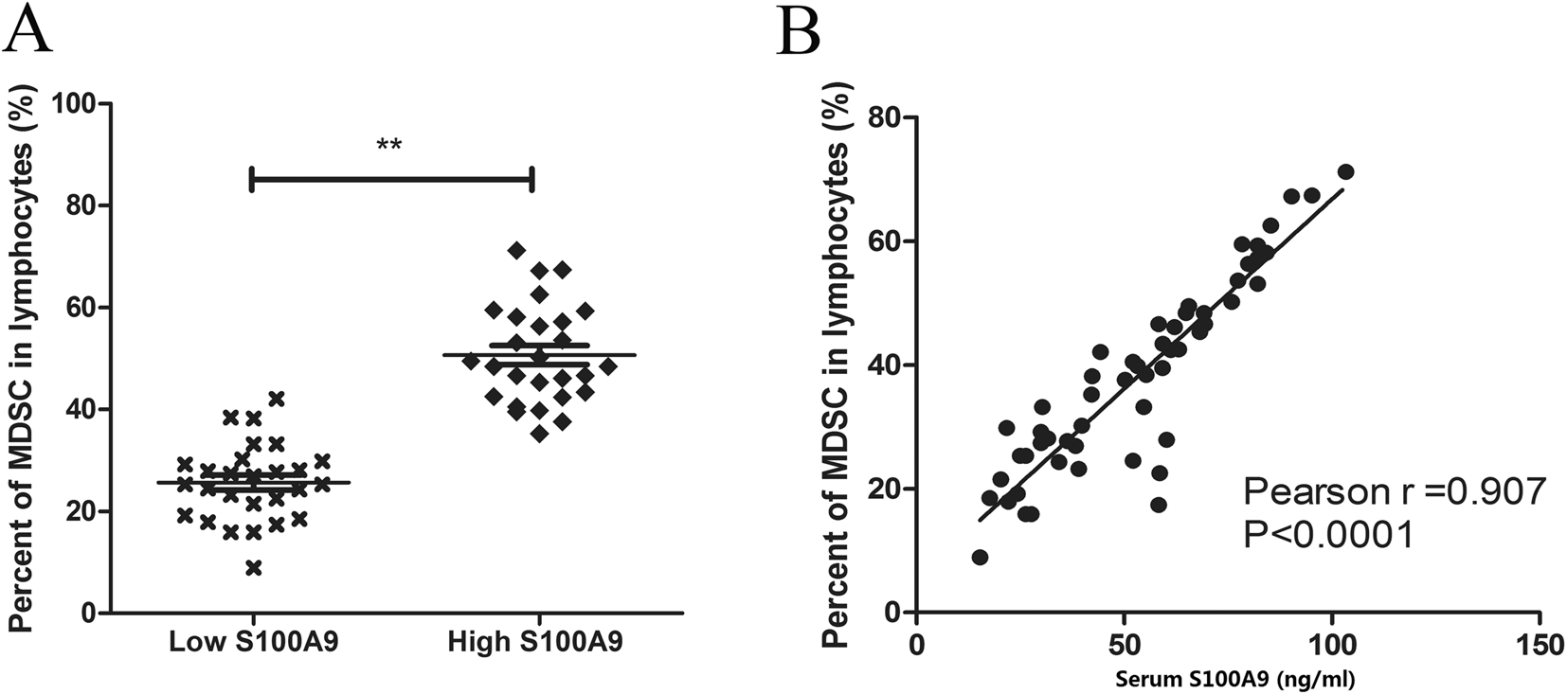
S100A9 was positively related to MDSCs in patients with NKTCL. (**A**) Patients with elevated levels of S100A9 (high-S100A9 group) possessed higher percentages of MDSCs than low-S100A9 group (*p* < 0.01). (**B**) Percentage of MDSCs in peripheral blood was positively correlated with serum levels of S100A9 (Pearson r = 0.907, *p* < 0.0001). **p < 0.01.

Myeloid-derived suppressive cells (MDSCs) are a group of immature cells negatively regulate immune response and lead to tumor progression. S100A9 was assumed as a marker of MDSCs, which could recruit MDSCs in tumor microenvironment. Thus, we analyzed the correlation between S100A9 and MDSCs in peripheral blood. Patients with elevated levels of S100A9 possessed higher percentages of MDSCs (*p* < 0.01) (Fig. 3A). Percentage of MDSCs in peripheral blood was positively correlated with S100A9 (Pearson r = 0.907, *p* < 0.0001) (Fig. 3B).

### Low concentration of S100A9 promotes proliferation of NKTCL cells

**Figure 4:**
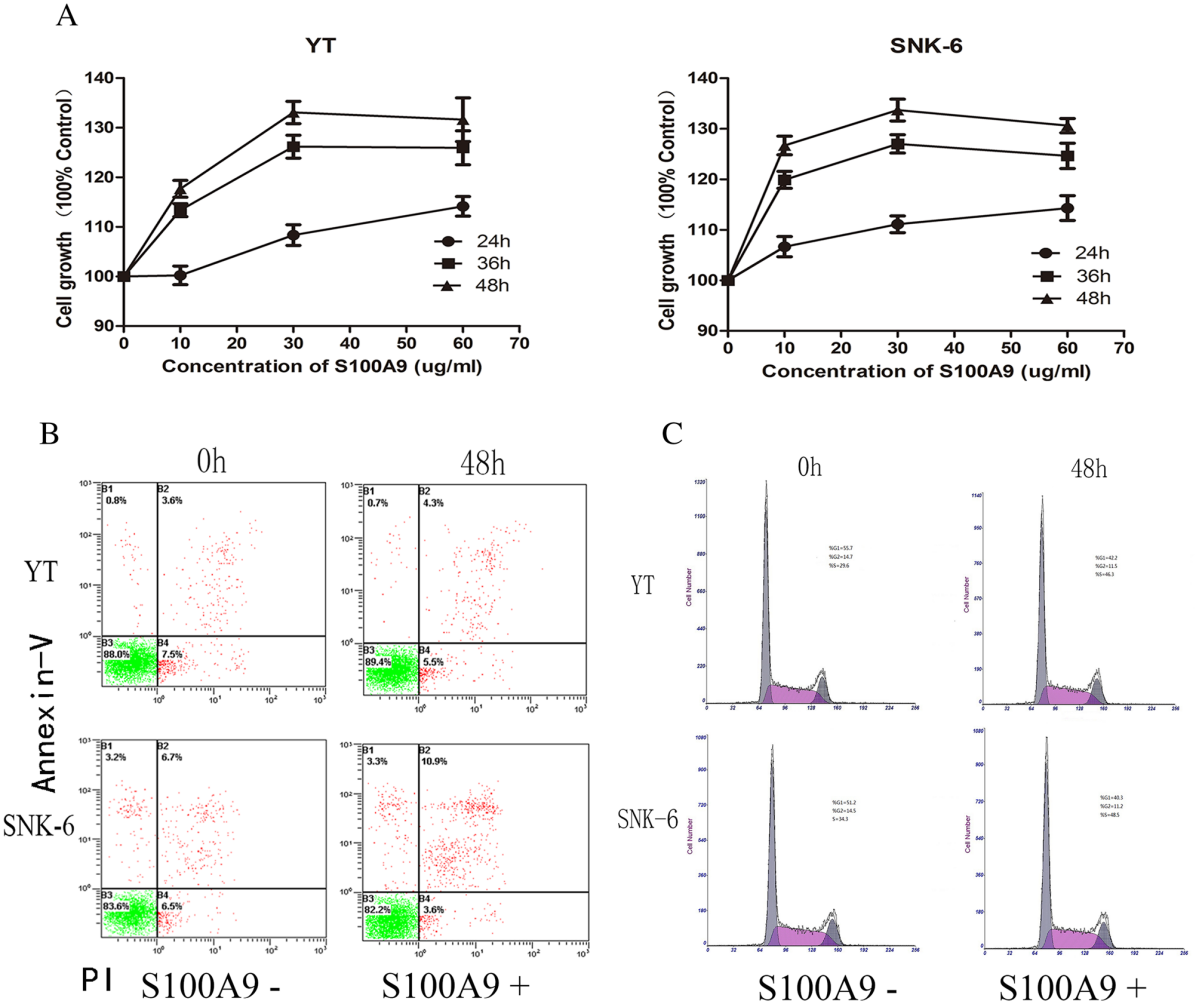
Effects of S100A9 on NKTCL cells (YT and SNK-6). (**a**) NKTCL cells were treated with different concentrations of S100A9 protein. CCK-8 assay showed low and mediate concentrations of S100A9 protein promoted proliferation of NKTCL cells. (**b**) NKTCL cells were exposure to S100A9 at the concentration of 30ug/mL for 48 h. Cell apoptosis and cell cycles of NKTCL cells were not affected by S100A9 protein.

To investigate the effects of extracellular S100A9 on NKTCL cells, we firstly constructed recombinant expression plasmid pET16b-S100A9 and obtained S100A9 recombinant protein. NKTCL cells (YT and SNK-6) were treated with different concentrations of S100A9 protein and cell viability was examined via CCK-8 assay at 24 h, 36 h and 48 h. Low (10–30 ug/mL) concentrations of S100A9 increased cell proliferation at 36 h and 48 h, while high concentration (60 ug/mL) did not further enhance cell proliferation and reached a plateau (Fig. 4a). Further results suggested that cell apoptosis and cycles were not affected by S100A9 (Fig. 4b, c). Thus, we selected concentration of 30ug/mL S100A9 protein for further functional research.

### S100A9 upregulates PD-L1 expression on NKTCL cells via activation of ERK1/2 signaling pathway

**Figure 5:**
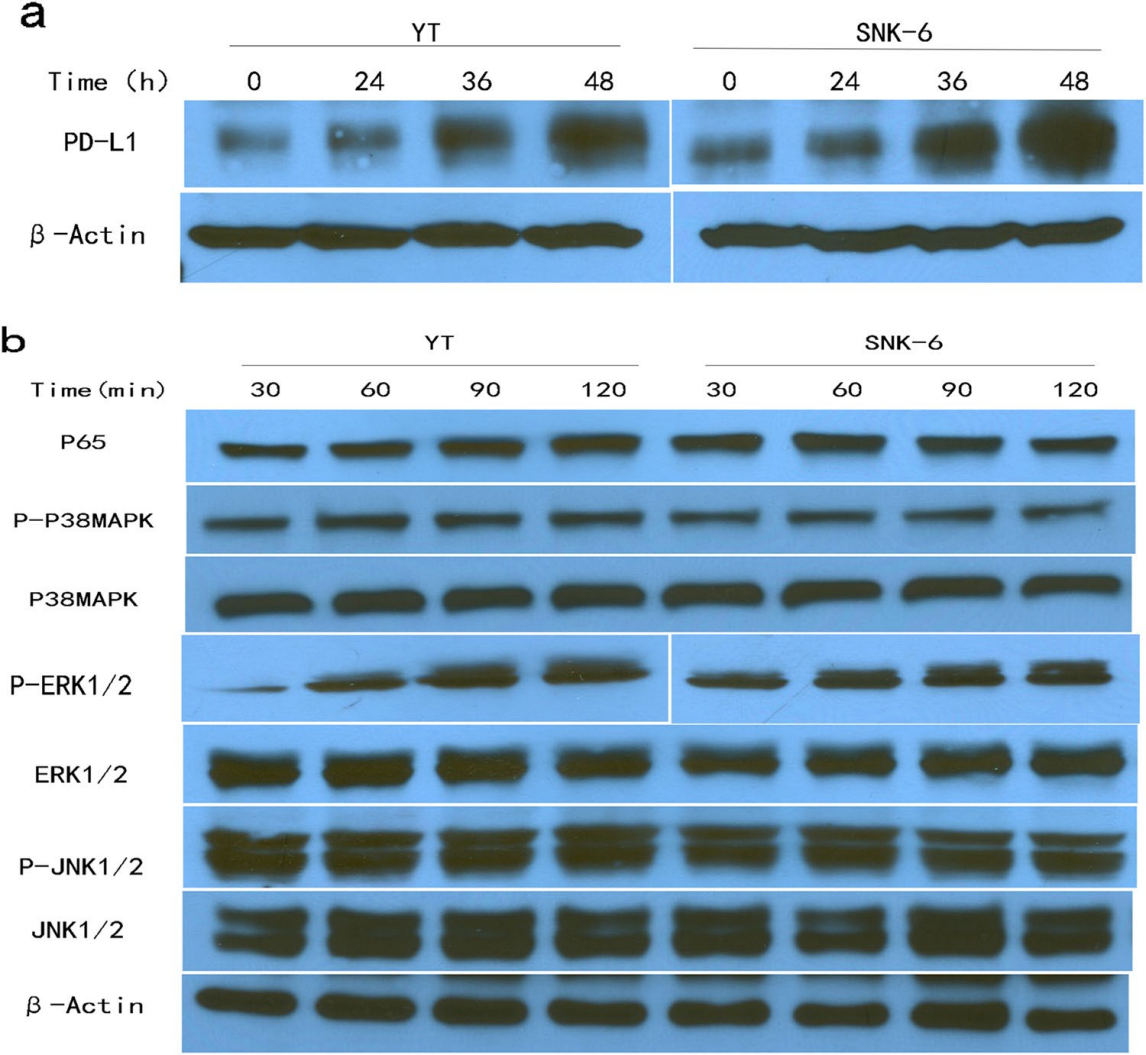
S100A9 upregulated PD-L1 expression and activated ERK1/2 signaling pathway Western blotting were performed to explore the effect of S100A9 on NKTCL cell lines. The blots were cut prior to hybridisation with antibodies during blotting. (**a**) NKTCL cells were exposure to S100A9 treatment for 24 h, 36 h and 48 h. PD-L1 expression on NKTCL cells were upregulated upon treatment with S100A9 protein. (**b**) Treatment with S100A9 activated ERK1/2 signaling pathway in NKTCL cells, while JNK1/2, p38 MAPK signaling were not changed.

**Figure 6:**
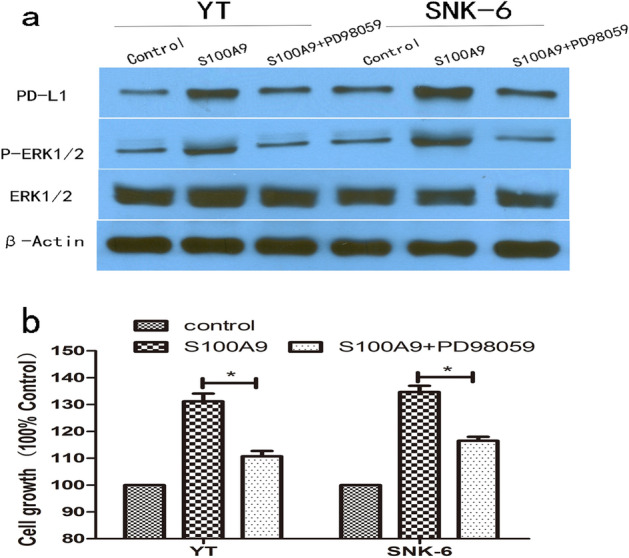
S100A9 induces PD-L1 expression and promotes cell proliferation via activation of ERK1/2 signaling in NKTCL cells. (**a**) ERK1/2 inhibitor (PD98059) decreased the upregulation of PD-L1 expression induced by S100A9. (**b**) ERK1/2 inhibitor (PD98059) also decreased cell proliferation promoted by S100A9 alone. *p < 0.05.

We examined the effects of S100A9 protein on PD-L1 expression in NKTCL cells. Upon treatment with S100A9, increased expression of PD-L1 protein were observed in both YT and SNK-6 cells (Fig. 5a). Moreover, we found that levels of phosphorylated ERK1/2 was enhanced when YT and SNK-6 cells were exposure to S100A9. Levels of other signaling proteins such as p65, p38MAPK, p-p38MAPK, JNK1/2, p-JNK1/2 were not changed (Fig. 5b). These datum showed that ERK1/2 signaling was also activated by S100A9. Co-treatment of NKTCL cells with ERK1/2 inhibitor (PD98059) reduced PD-L1 expression induced by S100A9 protein (Fig. 6a). Inhibition of ERK1/2 signaling with PD98059 also decreased proliferation of NKTCL cells promoted by S100A9 (Fig. 6b). These results suggested that the tumor growth and PD-L1 upregulation by S100A9 was mediated by activation of ERK1/2 signaling pathway.

### Serum S100A9 was an independent prognostic factor for NKTCL patients

**Figure 7:**
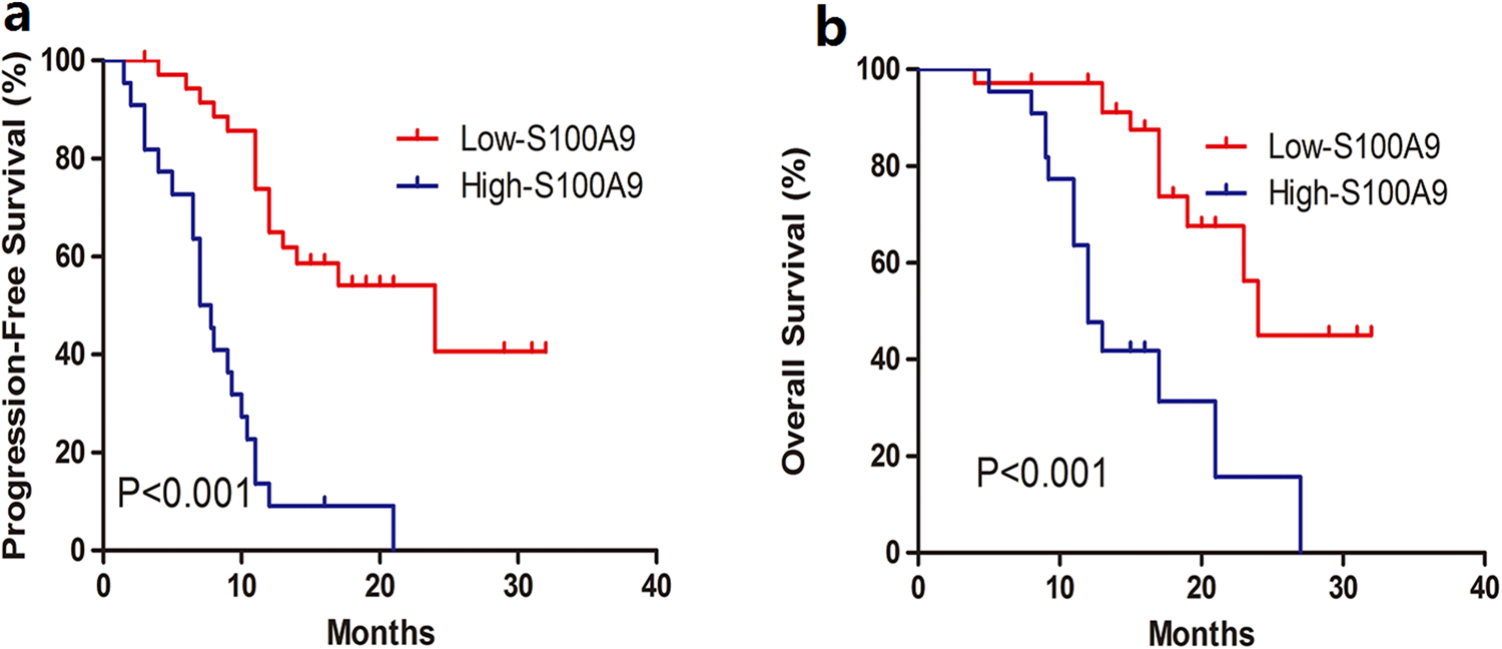
Survival analysis of patients according to the serum levels of S100A9. Patients with high S100A9 levels was associated with inferior PFS (*P* < 0.001) and OS (*P* < 0.001) than those with low S100A9 (Figure 7a and b).

Survival analysis was performed using the Kaplan–Meier method and the log-rank test. Multivariate analysis using Cox regression model was conducted. From the cox regression model analysis, we found that both serum S100A9 and PINK-E were independent prognostic factors for NKTCL patients (Table 2). Patients with high serum S100A9 levels was associated with inferior PFS (*p* < 0.001) and OS (*p* < 0.001) than those with low S100A9 (Fig. 7). These results indicated that elevated levels of S100A9 in serum was an independent adverse factor for NKTCL patients.

**Table 2:** Univariate and multivariate analysis of OS and PFS in NKTCL patients.

Variables	OS			PFS		
	Univariate analysis	Multivariate analysis		Univariate analysis	Multivariate analysis	
	*P*	RR (95% CI)	*P*	*P*	RR (95% CI)	*P*
Age > 60	0.125			0.065		
LDH > 245U/L	0.12			0.08		
Stage	0.05			0.024		
PINK-E > 2	0.02	4.32 (1.11–16.9)	0.036	0.006	11.1 (3.36–33.3)	< 0.001
EBV-DNA positive	0.08			0.067		
High-S100A9	< 0.001	7.14(2.78–18.8)	< 0.001	< 0.001	10 (4.18–21.7)	0.001

*LDH* lactate dehydrogenase, *EBV* Epstein-barr virus, *PINK-E* prognostic index of natural killer lymphoma-Epstein-barr virus.

## Discussion

In the present study, we conducted serum proteomic analysis and identified 199 dysregulated proteins in serum from NKTCL patients compared with healthy individuals. S100A9 was overexpressed in both serum and tumor tissue from NKTCL patients compared with healthy individuals. Moreover, high expression of S100A9 in tumor stroma was associated with advanced stage, poor response rate and early recurrence. In vitro study showed that extracelluar S100A9 protein promoted cell proliferation of NKTCL cells and enhanced PD-L1 expression via activation of ERK1/2 signaling. Our research firstly uncovered the role of S100A9 protein in inflammation-associated NKTCL. S100A9 could be served as a novel target in the treatment of NKTCL.

S100A9 is a member of the family of calcium-binding proteins, which is mainly released from myeloid cells. Previous studies demonstrated that S100A9 could active and enlarge inflammatory signaling^15^. In addition, S100A9 is deeply involved in cancer development and malignancy transformation. MDSC are a group of immunosuppressive cells which could inhibit T cell activation and lead to immune escape. S100A9 could recruit myeloid-derived suppressive cells (MDSC) in tumor microenvironment through interaction with RAGE or TLR on MDSC^16^. Knock out of S100A9 delayed tumor growth and decreased MDSC in tumor tissues^17^. S100A9 also inhibited differentiation of dendritic cells and macrophages to impair immune response^18^. Moreover, extracelluar S100A9 protein enhanced the proliferation of hepatocellular carcinoma cells and breast cancer cells and contribute to invasive phenotype^19,20^. Elevated expression of S100A9 in oral cancer not only increase tumor invasion but also induce macrophage recruitment^21^. However, Laouedj M found that S100A9 could induce differentiation and maturation of acute myeloid leukemia cells^22^.

As an immune suppressive molecule, PD-L1 is upregulated in various cancer cells and enables cancer cells to escape from host immune attack. The interaction of PD-L1 with its receptor PD-1 lead to apoptosis and dysfunction of immune cells. High expression of PD-L1 in cancer patients is frequently associated with poor prognosis^23^. Currently, inhibitors targeting PD1/PD-L1 axis have been developed and show promising efficacy in clinical. However, the response rate is low and few patients could benefit due to primary or acquired resistance^24^. Considerable efforts have been made to understand the mechanisms of regulation of PD-L1, which aims to improve the outcomes of anti-PD-L1/PD-1 therapy. Certain transcriptional factors such as STAT3, HIF-αcould bind the promoter of PD-L1 and enhance its expression. Activation of MAPK and PI3K/AKT signaling pathways have also been demonstrated to increase PD-L1 expression^25^. Recent researches indicated that amplification of chromosome 9p24.1 gave rise to activation of JAK2 signaling and overexpression of PD-L1 expression in breast cancer and lymphoid malignancies^26,27^. However, what we are interested is how inflammation regulates PD-L1 expression. Several inflammatory factors such as IFN-γ, IL-10 and TNF-αhave been reported to be responsible for PD-L1 expression^14,28,29^. In the present study, we firstly demonstrated that inflammatory factor S100A9 could induce PD-L1 expression via ERK1/2 signaling activation.

In summary, the relationships between inflammation and malignant diseases have been widely studied. As an EBV infection associated malignancy, the exact role of inflammation in tumorigenesis of NKTCL is largely unknown. Our results uncovered the role of S100A9 in inflammation-associated NKTCL. However, the role of S100A9 in MDSC-mediated immune escape in NKTCL need to be further explored. Currently, the difficulties in establishing mouse model of NKTCL restrict our study of S100A9 in vivo. Further study we will employ S100A9 knockout mice to examine the function of S100A9 in immunosuppressive microenvironment during tumor growth of NKTCL.

## Materials and methods

### Patients and sample collection

Informed consent was obtained from all individual participants included in the study. The patients provided written consent to the results for publication. Serum samples from 16 newly diagnosed NKTCL patients and 16 healthy volunteers were collected for comparative proteomic analysis. Serums were stored at  − 80 °C until further experiment. For validation of candidate proteins, serums from 45 healthy volunteers and 71 NKTCL patients were collected. Paraffin embedded sections from NKTCL tumor tissues and normal nasal mucosa tissues were also collected from department of pathology in our hospital. All experimental procedures were approved by the ethics committee of the First Affiliated Hospital of Zhengzhou University, and performed according to approved regulations.

### Cell lines and cell culture

The human NKTCL cell line SNK-6 was a gift from Professor Norio Shimizu (Chiba University Hospital, Chiba, Japan), and cultured in RPMI 1640 medium containing 15% heat-inactivated human serum, 700 U/mL interleukin-2 (IL-2). The human NKTCL cell line YT was preserved in our lab and cultured in RPMI 1640 medium containing 15% heat-inactivated FBS.

### LC–MS/MS: proteomic analysis

iTRAQ and LC–MS/MS (liquid chromatography tandem mass spectrometry) based proteomic analysis were performed^30^. Briefly, serum proteins were digested into peptides. Strong cation exchange (SCX) chromatography was conducted to reduce sample complexity. The peptide fractions were collected and analyzed through mass spectrometry. Tandem mass spectra was searched against database to identify peptides and proteins. All reported data were based on 99% confidence for protein identification as determined by false discovery rate (FDR) ≤ 1%. Gene ontology (GO) annotation was conducted for the differentially expressed proteins, and they were also mapped to signaling pathways in KEGG.

### Enzyme-linked immunosorbent assay (ELISA)

S100A9 was selected as candidate protein for further validation in another cohorts of NKTCL patients and healthy individuals via ELISA. Serum levels of S100A9 were measured following the procedures provided by the commercially available ELISA Kit (Uscn Life Science, Wuhan, China).

### Immunohistochemistry (IHC) assay

Sections from paraffin embedded tissues were incubated and followed by deparaffinage and hydration. Antigen retrieval was conducted and endogenous peroxidase was blocked by 3% H_2_O_2_. After washed three times with PBS, the sections were blocked with goat serum and incubated with primary antibody at 4 ℃ overnight. Secondary antibody was added and incubated for 30 min. DAB reagent was used to visualize the reaction. Representative images were captured under Nikon light microscope. Expression levels of S100A9 protein were calculated as intensity score multiplies percentage score.

### Cell viability via CCK-8 assay

To evaluate cell proliferation, Cell Counting Kit-8 (CCK-8) assay was performed according to manufacturer’s instructions. Briefly, cells were seeded into 96-well plates (3.6 × 10^4^ cells/180 μL) with or without treatment. After incubation for a certain period, 20 μL of CCK-8 reagent was added to each well. Then, the plates were placed into the humidified incubator again at 37℃ for 2–4 h. The absorbance was measured on a microplate reader at 450 nm wavelength of light.

### Western blotting

Cell protein extracts were diluted with 5 × SDS loading buffer and heated at 95 ℃. Equal amounts of proteins were loaded onto SDS–polyacrylamide gels. Proteins were electrophoresed and transferred to nitrocellulose membranes at constant 200 mA. Membranes were blocked with 5% nonfat dry milk followed by incubation with primary antibodies at 4 ℃ overnight. The blots were cut prior to hybridisation with antibodies during blotting. After that, membranes were exposed to HRP-Conjugated secondary antibodies (1:9000 dilution) for 1 h. Bands were visualized through Chemi Doc XRS + imaging system (Bio-rad Company, USA).

### Statistical analysis

SPSS software 17.0 was employed to perform statistical analysis. All numerical datas were presented as mean ± standard deviation of the mean. Standard two-tailed Student’s t-test was to examine statistical difference, which was considered to be significant if *p* value < 0.05.

### Ethical approval

All experimental procedures were approved by the ethics committee of the First Affiliated Hospital of Zhengzhou University, and performed according to approved regulations.

## Supplementary Information

Supplementary Information.
