# Supplementary material for: Overexpression of S100A9 in tumor stroma contribute to immune evasion of NK/T cell lymphoma and predict poor response rate
Source: Sci Rep. 2021 May 27;11:11220. doi: 10.1038/s41598-021-90794-3 (PMC8160340; doi:10.1038/s41598-021-90794-3)
Supplement: Supplementary file 1 — Supplementary Information. [file 41598_2021_90794_MOESM1_ESM.docx]

**Title page:**

**Overexpression of S****100A9 in tumor stroma contribute to immune evasion of NK/T cell lymphoma and predict poor response rate**

Zhiyuan Zhou^1, 2,#^, Xinfeng Chen ^1,3,#^, Zhaoming Li^1, 2^, Xinhua Wang^1^, Mingzhi Zhang^1, 2★^

^1^ Department of Oncology, The First Affiliated Hospital of Zhengzhou University, No.1 Jianshe East Road, Zhengzhou, 450000, China

^2^ Institute of Clinical Medicine, The First Affiliated Hospital of Zhengzhou University, No.1 Jianshe East Road, Zhengzhou, 450000, China

^3^ Biotherapy Center, The First Affiliated Hospital of Zhengzhou University, No.1 Jianshe East Road, Zhengzhou, 450000, China

^#^Zhiyuan Zhou and Xinfeng Chen contributed equally to this paper.

**^★^Corresponding author：**Prof. Mingzhi Zhang, Email: mingzhi_zhang1@163.com; Tel: +86-13838565629; Fax: +86-0371-66295562; Department of Oncology, The First Affiliated Hospital of Zhengzhou University, No.1 Jianshe East Road, Zhengzhou, 450000, China

**Supplemental materials：**

**Table SI Representative proteins differentially expressed in serum between NKTCL patients and healthy individuals.**

|  |  | | | |
| --- | --- | --- | --- | --- |
| **Name** | **Levels** | | **Ratios** | **FDR** |
|  | **NKTCL**  **patients** | **Healthy individuals** | **NKTCL patients /Healthy individuals** |  |
| **Upregulated proteins** |  |  |  |  |
| Conserved oligomeric Golgi complex subunit 3 (Fragment) | 1.57988576 | 0.10246283 | 15.4191115 | 0.005591241 |
| Heat shock protein HSP 90-beta | 2.4690229 | 0.46705976 | 5.28631044 | 0.028360458 |
| Ferritin light chain | 2.43431005 | 0.58584374 | 4.15522076 | 0.000952182 |
| Keratin, type I cytoskeletal 17 | 2.21877301 | 0.57902444 | 3.831916 | 0.007255994 |
| Flap endonuclease 1 (Fragment) | 1.11026689 | 0.35291139 | 3.14602172 | 0.028504972 |
| Ferritin | 2.02175285 | 0.74845954 | 2.70121862 | 0.007238256 |
| 14-3-3 protein epsilon | 1.58043351 | 0.59769112 | 2.64423122 | 0.00257625 |
| Proteasome subunit beta type-2 | 1.81161169 | 0.69658164 | 2.60071697 | 0.011065553 |
| PTX3 | 1.19753674 | 0.50469829 | 2.37277749 | 0.000853598 |
| Protein S100-A9 | 1.44016135 | 0.61535242 | 2.34038463 | 0.002569889 |
| MHC class I antigen (Fragment) | 1.30923225 | 0.56516929 | 2.31653113 | 0.010332082 |
| Blood group Rh(CE) polypeptide | 1.72342478 | 0.7505662 | 2.29616626 | 0.021485383 |
| 14-3-3 protein zeta/delta | 1.51214696 | 0.66105472 | 2.28747623 | 0.010332082 |
| Alpha-1-acid glycoprotein 1 | 1.20887627 | 0.53505536 | 2.25934802 | 0.003035581 |
| **Downregulated proteins** |  | | | |
| armadillo repeat containing 2 | 0.98522416 | 3.56178656 | 0.27660955 | 0.045511293 |
| Platelet factor 4 | 0.40440113 | 1.37200251 | 0.29475247 | 0.010657236 |
| Antithrombin | 0.9094889 | 2.2559989 | 0.40314244 | 0.013360368 |
| Chromogranin-A | 0.56595365 | 1.30637122 | 0.43322574 | 0.012160264 |
| scavenger receptor cysteine-rich domain-containing protein | 0.91518854 | 1.89134363 | 0.48388274 | 0.03223241 |
| Reticulon 4 receptor-like 2 | 0.88457584 | 1.66712413 | 0.53059987 | 0.03223241 |
| Platelet basic protein | 0.6581673 | 1.19712036 | 0.54979209 | 0.006989596 |
| Amyloid beta A4 protein | 0.71572154 | 1.29836289 | 0.55124923 | 0.007221976 |
| Hepatocyte growth factor-like protein | 0.7305642 | 1.29098998 | 0.56589456 | 0.022287357 |
| C4b-binding protein alpha chain | 0.85956764 | 1.50713551 | 0.57033202 | 0.033114566 |
| Gelsolin | 0.72504877 | 1.26188417 | 0.57457633 | 0.008369854 |
| Plasma serine protease inhibitor | 0.76174275 | 1.32077707 | 0.57673832 | 0.00196482 |
| Protein S (Fragment) | 1.02088161 | 1.71625775 | 0.59483001 | 0.031919355 |

**
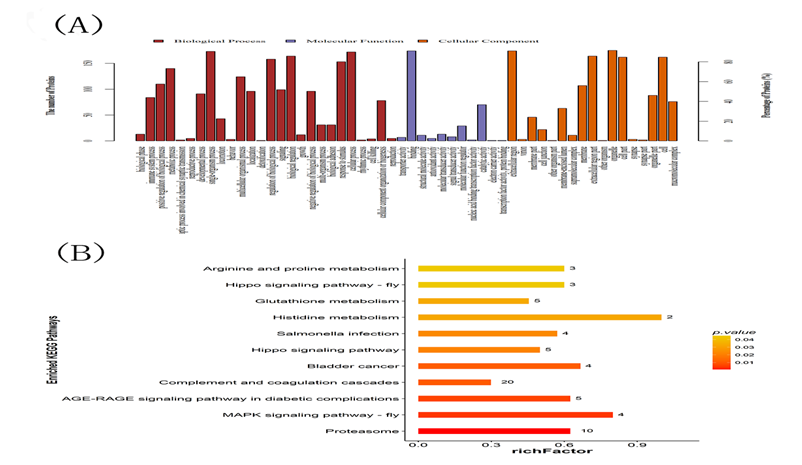
**

**Figure SI.** Bioinformatics analysis of the differentially expressed proteins between NKTCL patients and healthy individuals. (A) Gene ontology (GO) annotation indicated that the dysregulated proteins were involved in the processes of immune system and biological regulation. (B) KEGG pathway analysis showed that the dysregulated proteins were enriched in proteasome pathway, MAPK and AGE-RAGE signaling pathway.

**Preparation of recombinant S100A9 protein**

Recombinant expression plasmid pET16b-S100A9 was firstly constructed. The whole coding sequence of human S100A9 gene was synthesized and connected with pET16b vector by T4 DNA ligase. The recombinant plasmid was validated by enzyme digestion and DNA sequencing. Then, pET16b-S100A9 was transformed into Rosetta2 cells, and expression of recombinant protein was induced by IPTG, purified with Ni-NTA agrose and identified by SDS-PAGE and western blot.

**
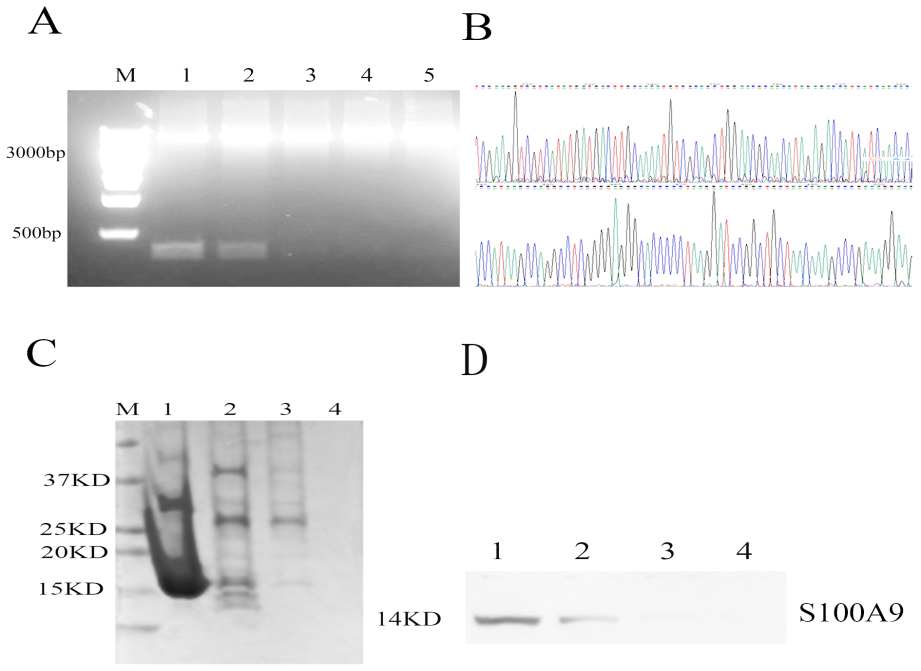
**

**Figure SII.** Preparation of S100A9 recombinant protein. (A and B) Validation of recombinant expression plasmid pET16b-S100A9 by enzyme digestion and DNA sequencing. (C) Identification of S100A9 recombinant protein through SDS-PAGE and western blot.

**Figure 5:**


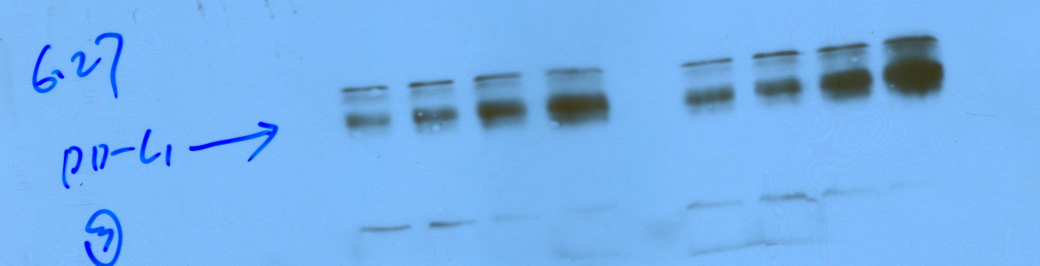


PD-L1

**
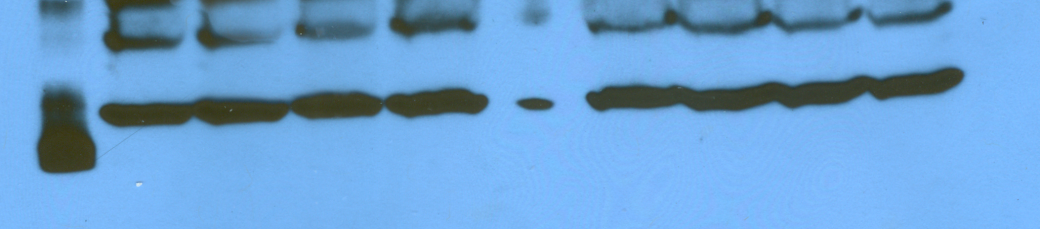
**

β-ACTIN

**
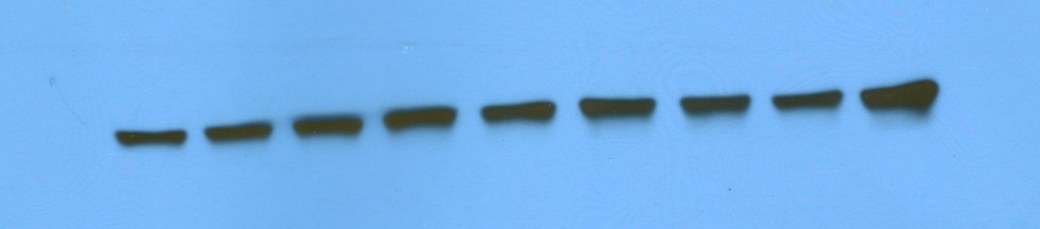
**

P65

**
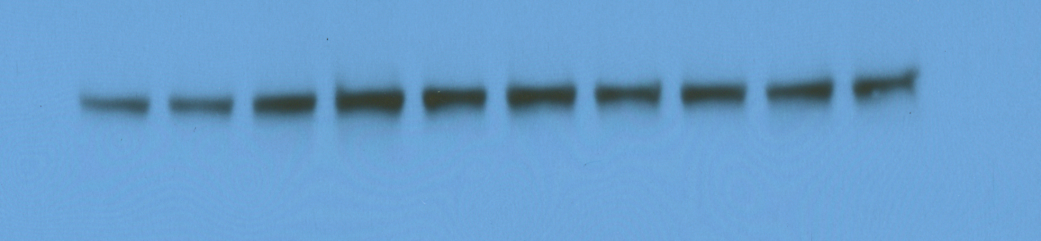
**

P-P38MAPK

**
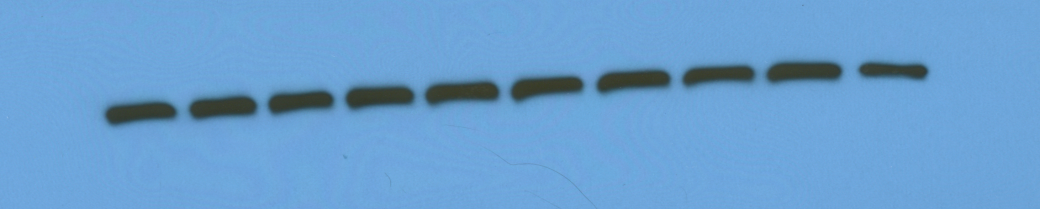
**

P38MAPK

**
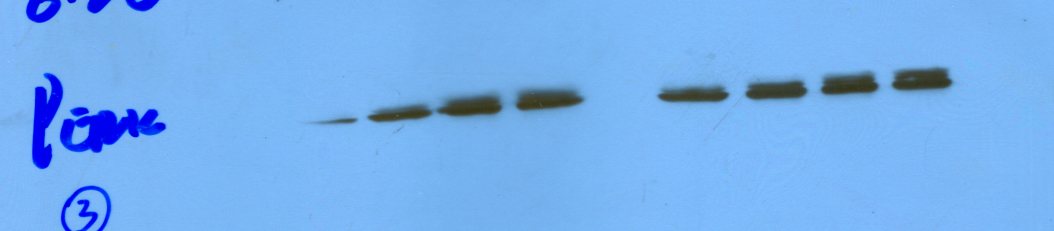
**

P-ERK1/2

**
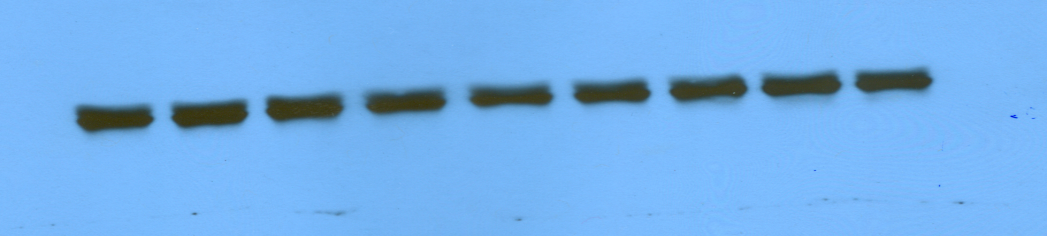
**

ERK1/2

**
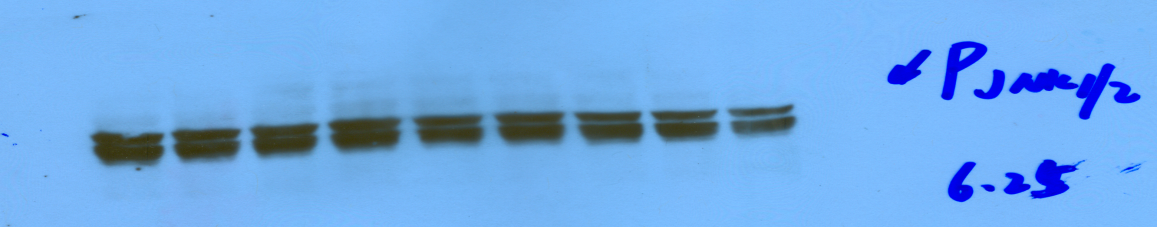
**

P-JNK1/2

**
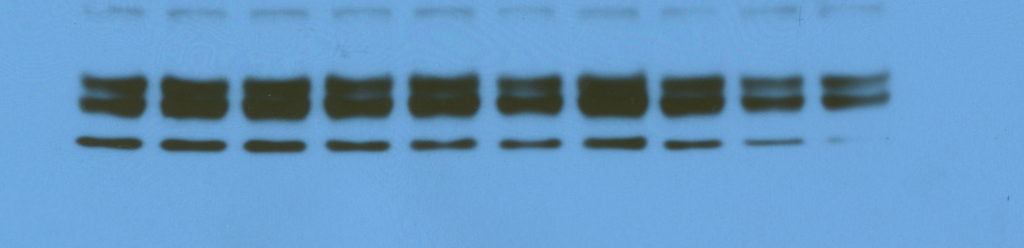
**

JNK1/2

**
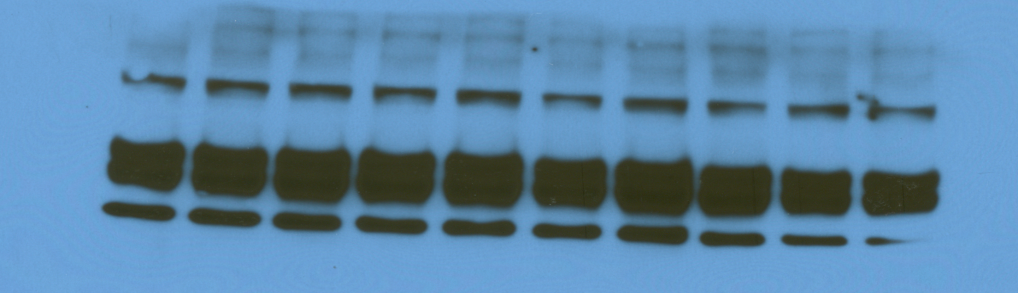
**

β-ACTIN

**Figure 6:**

**
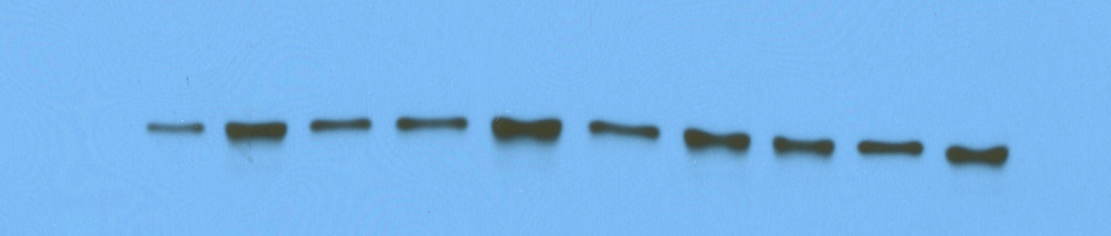
**

PD-L1

**
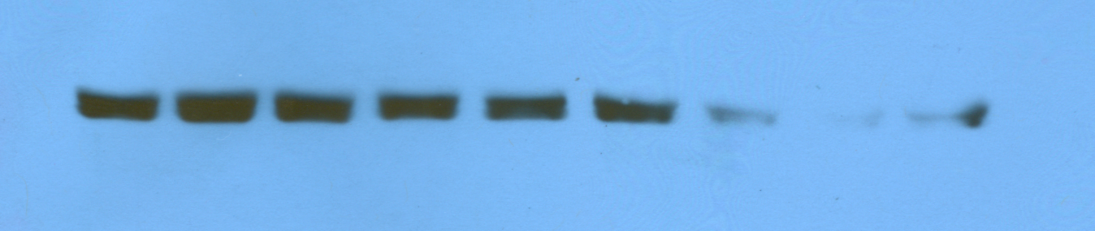
**

ERK1/2

**
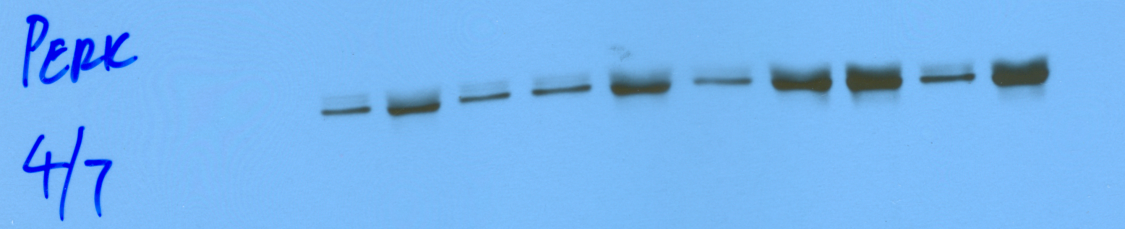
**

P-ERK1/2

**
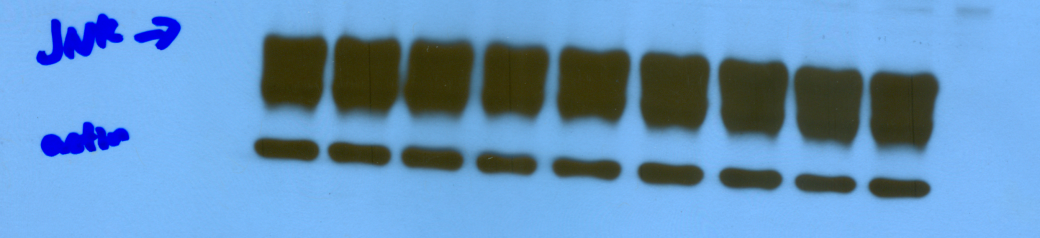
**

β-ACTIN

**Figure SII.:**

**
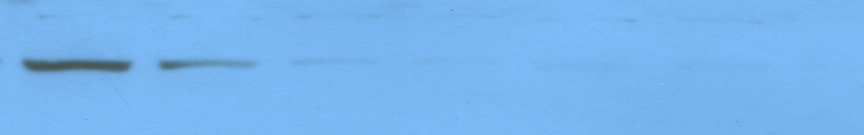
**

S100A9

**Figure 5:**

**
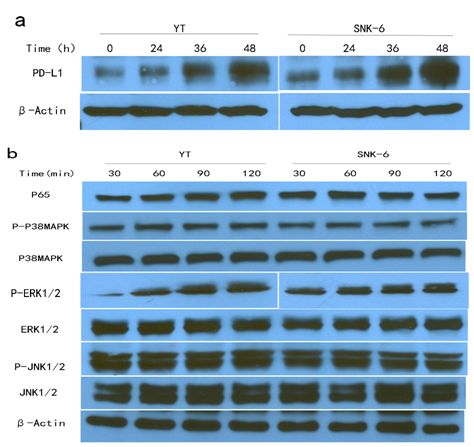
**

**Dear editor:**

**Thank you very much for your suggestion. Following your suggestion, I have used white spacing to present Figure 5a-actin, 5b-p-ERK1/2.** **I also added the sentence “The blots were cut prior to hybridisation with antibodies during blotting” in the methods part. If you have any questions please feel free to contact me.**

**Best wishes to you!**
